# Idiopathic pre-capillary pulmonary hypertension in patients with end-stage kidney disease: effect of endothelin receptor antagonists

**DOI:** 10.1007/s10157-016-1344-y

**Published:** 2016-10-19

**Authors:** Masato Nishimura, Toshiko Tokoro, Satoru Yamazaki, Tetsuya Hashimoto, Hiroyuki Kobayashi, Toshihiko Ono

**Affiliations:** 1 Cardiovascular Division, Toujinkai Hospital, 83-1, Iga, Momoyama-cho, Fushimi-ku, Kyoto, 612-8026 Japan; 2 Department of Nephrology, Toujinkai Hospital, Kyoto, Japan; 3 Department of Urology, Toujinkai Hospital, Kyoto, Japan

**Keywords:** Endothelin receptor antagonist, End-stage kidney disease, Pre-capillary, Pulmonary hypertension, Right heart catheterization

## Abstract

**Background:**

We examined the prevalence, prognosis, and effect of endothelin receptor antagonists on survival in end-stage kidney disease patients with idiopathic pre-capillary pulmonary hypertension.

**Methods:**

We investigated 1988 end-stage kidney disease patients in Toujinkai Hospital from January 1, 2001 to December 31, 2014. Pulmonary hypertension was screened by symptoms (dyspnea, hypotension, or near syncope) and echocardiography, and diagnosed by computed tomography with enhancement, pulmonary flow scintigraphy, and right heart catheterization.

**Results:**

Fifteen patients (67 ± 11 years; 12 women and 3 men) were diagnosed as idiopathic pre-capillary pulmonary hypertension; mean pulmonary arterial pressure, pulmonary vascular resistance, or pulmonary artery wedge pressure were 55 ± 11 mmHg, 7.5 ± 2.9 Woods units, or 12 ± 2 mmHg, respectively. Of the 15 patients, 14 received hemodialysis, and 1 was in a pre-dialysis stage. Patients were followed through December 31, 2015, and 11 died of heart failure; their mean survival time was 26.4 ± 21.0 months. Endothelin receptor antagonists were used for 11 patients, and mean survival times were 57.3 ± 12.1 months in patients with endothelin receptor antagonists and 7.5 ± 2.1 months in those without. In the Kaplan–Meier analysis, heart failure death-free survival rates were higher in patients with endothelin receptor antagonists than in those without (*P* < 0.001); 100 versus 25 % at one year and 71 versus 0 % at 3 years.

**Conclusion:**

The prognosis of idiopathic pre-capillary pulmonary hypertension seems to be poor in end-stage kidney disease patients. Administration of endothelin receptor antagonists might improve the survival by inhibiting heart failure death.

*Registration of clinical trials* This study was registered to the ClinicalTrials.gov (https://clinicaltrials.gov/): protocol identifier, NCT02743091.

## Introduction

Pulmonary hypertension (PH) is a rare cardiovascular disease with progressive and fatal features [1]. PH is classified into 5 groups, and the prevalence of group 1, pulmonary arterial hypertension (PAH), including idiopathic and heritable types, is 5 to 15 cases per one million adults, and the median survival was reportedly 3 years in those for whom treatments had not been administered [2, 3]. PH found in patients with end-stage kidney disease (ESKD) is classified into group 5, because the pathogenesis and clinical characteristics have not been clarified [1]. The prevalence of PH in patients with ESKD was reportedly around 17–56 % based on echocardiographic studies [4–10]. Echocardiography is useful for screening PH, but it may be misleading in the assessment of patients with suspected PH, especially when an inadequate tricuspid regurgitation jet is over-estimated [11–13]. Furthermore, echocardiography could not discriminate between pre- and post-capillary PH. Recently, Pabst et al. [14] reported that pre-capillary PH was identified in 4 of 31 (12.9 %) hemodialysis patients with dyspnea evaluated by right heart catheterization (RHC), whereas post-capillary PH was found in 20/31 (64.5 %) patients. ESKD patients are likely to have post-capillary PH mainly due to volume overloading or left heart diseases, such as coronary artery disease. Therefore, evaluation by RHC is essential for diagnosis of pre-capillary PH in ESKD patients.

PH in ESKD patients has now begun to be focused on in the field of nephrology; however, clinical features of idiopathic pre-capillary PH are still unclear, and no study has been reported on the treatment of PH in this population. In this study, we investigated the prevalence and prognosis of idiopathic pre-capillary PH in ESKD patients of our hospital, and examined the effect of endothelin receptor antagonists (ETAs) on survival and inhibition of the heart failure death.

## Methods

This is a retrospective analysis performed in a single center. A total of 1988 ESKD patients who had received medical treatment at pre-dialysis stage [Chronic kidney disease (CKD) Stage 5] or had been undergoing maintenance hemodialysis in Toujinkai Hospital from January 1, 2001 to December 31, 2014 were investigated regarding PH.

### Patients

**Fig. 1:**
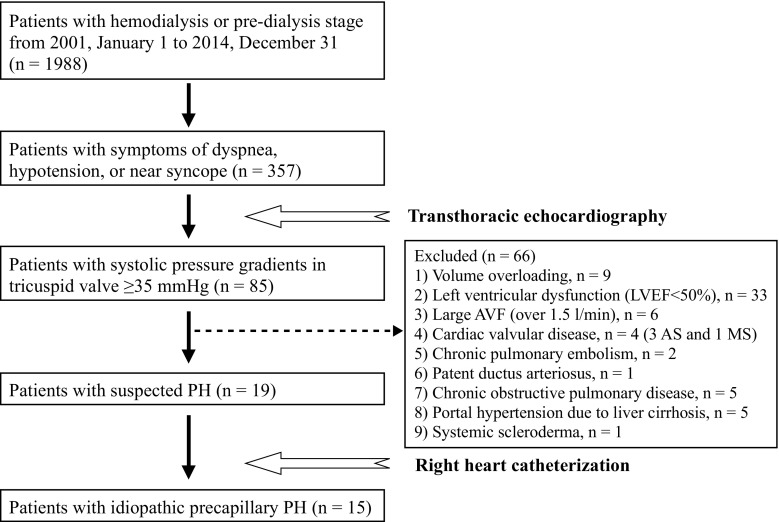
Flow chart of patient selection. The levels of dyspnea were World Health Organization functional class ≥ II, and hypotension was in a state of systolic blood pressure consistently below 100 mmHg. *LVEF* left ventricular ejection fraction, *AVF* arteriovenous fistula, *AS* aortic valvular stenosis, *MS* mitral valvular stenosis, *PH* pulmonary hypertension

A flow chart of patient selection is shown in Fig. 1. Of 1988 patients treated with ESKD, medical consultation was sought at the cardiovascular division of Toujinkai Hospital regarding 357 patients (270 hemodialysis and 87 CKD stage 5) that had symptoms of dyspnea (World Health Organization functional class ≥II), consistent hypotension (systolic blood pressure below 100 mmHg), or near syncope. Transthoracic echocardiography was performed for these 357 patients; it was performed after the last hemodialysis session of the week in hemodialysis patients. We chose 85 patients (66 hemodialysis and 19 CKD stage 5) with systolic pressure gradients (PGs) in tricuspid valve ≥35 mmHg for further examination according to the guidelines of the Japanese Circulation Society (1999–2000). Secondary or other types of PH were ruled out by echocardiography of the heart, abdomen and veins of the lower limbs, computed tomography of the chest and abdomen with enhancement, pulmonary function test and flow scintigraphy, myocardial scintigraphy (fatty acid imaging) [15], and blood laboratory test for collagen diseases. Coronary angiography was performed when echocardiography or myocardial scintigraphy indicated myocardial ischemia. Chronic obstructive pulmonary disease was defined by percent predicted forced expiratory volume in one second <60 %. Of 85 patients, 66 were excluded due to the reasons described in Fig. 1. Obstructive coronary artery disease was identified in 27 of 33 patients with left ventricular ejection fraction <50 %. In the results, RHC was performed for 19 patients (18 hemodialysis and 1 CKD stage 5), and idiopathic pre-capillary PH was diagnosed in 15 patients according to the criteria described below.

### RHC

RHC was undergone within 12 h after the last hemodialysis session of the week in a state of fasting after dialysis using a 6F balloon-tipped flow-directed Swan-Ganz catheter (Argon Critical Care Systems Singapore Pte. Ltd). In patients of pre-dialysis stage, RHC was performed in a state of fasting over 12 h. Pre-capillary PH was defined as mean pulmonary arterial pressure (PAP) ≥25 mmHg, pulmonary vascular resistance (PVR) >3 Woods units, and pulmonary artery wedge pressure (PAWP) ≤15 mmHg; PAP ≥ 25 mmHg and PAWP > 15 mmHg were diagnosed as post-capillary PH. PVR was obtained from the formula as follows: (mean PAP–PAWP)/cardiac output. Cardiac output was estimated by the thermo-dilution method, and cardiac index was determined by cardiac output/body surface area (m^2^).

### Medications

Antiplatelet drugs (aspirin 100 mg/day) and oral prostaglandin I_2_ (beraprost sodium, 180 µg/day) were administered to all patients diagnosed as pre-capillary PH. ETAs (Bosentan, ambrisentan, or macitentan) were used in 11 of 15 patients. Other drugs, such as cyclic GMP phosphodiesterase type 5 inhibitors and intravenous prostaglandin I_2_, were not used in our institute, because they exhibit stronger blood pressure reduction than ETAs.

### Statistical analysis

Values were expressed as the mean ± SD in the text and median value (maximum, minimum) in the tables. The means of continuous variables were compared using Mann–Whitney’s *U* test. Categorical data were analyzed using the *χ* ^2^ test. Heart failure death-free survival rates by use/non-use of ETAs were assessed using the Kaplan–Meier method and the log-rank test. Prognosis was assessed using the Cox proportional hazards models. *P* values of <0.05 were considered significant. All statistical analyses were performed using IBM SPSS Statistics software, version 23.

## Results

Of 1988 patients with ESKD, 15 patients were diagnosed as idiopathic pre-capillary PH; the incidence was 0.75 % of all patients (15/1988), 4.2 % of patients with symptoms of dyspnea, hypotension, or near syncope (15/357), and 17.6 % of patients with systolic PG in tricuspid valve ≥35 mmHg (15/85). Of 19 patients who had undergone RHC, 3 were post-capillary PH and 1 was mean PAP <25 mmHg. Clinical features of these 15 patients are described in Table 1. One patient (patient number 3) was in a CKD stage 5, and the other 14 patients underwent maintenance hemodialysis. The number of women was more than that of men (12/15), and the proportion of those with diabetes mellitus was 0.2. Systolic blood pressure before dialysis at the onset of PH was below 90 mmHg in all patients. All 15 patients had vascular access, such as arteriovenous graft (patient number 3) or arteriovenous fistula (AVF) (other patients), in upper limb, and blood flow of vascular access was below 1 l/min, evaluated by Doppler ultrasound. Although the mean durations of the onset of PH after making vascular access were 91 ± 123 months, pre-capillary PH was diagnosed within 6 months of making vascular access in 8 patients (53 %).

**Table 1:** Clinical characteristics of patients at the onset of pulmonary hypertension

Patient number	Age (years)	Dialysis duration (months)	Female gender (1 = yes, 0 = no)	Diabetes mellitus (1 = yes, 0 = no)	Body mass index (kg/m^2^)	Systolic blood pressure (mmHg)	Duration after making vascular access (months)
1	73	4	1	0	18.1	78	3
2	46	6	0	1	26.3	82	4
3	71	0	1	0	17.7	66	3
4	47	59	1	1	22.4	72	4
5	71	136	1	1	24.6	88	138
6	53	420	1	0	18.4	56	5
7	62	396	0	0	18.9	84	400
8	76	214	1	0	18.6	76	220
9	64	344	1	0	19.4	76	6
10	83	135	1	0	18.2	68	135
11	75	110	1	0	23.1	86	16
12	76	260	1	0	17.7	82	4
13	69	519	1	0	19.5	78	6
14	70	436	0	0	23.6	74	250
15	69	159	1	0	23.3	82	166
Median (maximum, minimum)	70 (83, 46)	159 (519, 0)			19.4 (26.3, 17.7)	78 (88, 56)	6 (3, 400)

### Hemodynamic measurements

The systolic PGs in tricuspid valve evaluated by echocardiography were extremely high in all 15 patients, and left ventricular ejection fraction was within normal range. In RHC, mean PAP ranged from 27 to 57 mmHg, PVR 3.1 to 14.4 Woods units, and PAWP 7 to 14 mmHg. Transpulmonary pressure gradient was ≥15 mmHg in all patients. Cardiac index was lower than 2.5 l/min/m^2^ in 4 patients (Table 2).

**Table 2:** Hemodynamic measurements in transthoracic echocardiography (TTE) and right heart catheterization (RHC)

Patient number	TTE		RHC						
	Systolic PG in tricuspid valve (mmHg)	LVEF (%)	Systolic PAP (mmHg)	Mean PAP (mmHg)	PAWP (mmHg)	RAP (mmHg)	PVR (Woods units)	TPG (mmHg)	CI (l/min/m^2^)
1	88	76	75	50	14	6	14.4	36	1.7
2	68	74	58	44	12	4	9.5	32	2.3
3	60	76	68	42	7	7	9.2	35	2.6
4	72	80	75	57	10	4	9.4	47	3.3
5	89	68	55	40	14	8	6.1	26	2.9
6	59	78	47	34	14	2	7.7	20	1.7
7	75	70	62	44	13	8	9.1	31	2.8
8	84	63	48	33	14	9	5.1	19	2.5
9	100	72	68	37	9	4	6.2	28	3.0
10	63	76	65	38	12	5	10.4	26	1.7
11	109	77	54	36	12	2	6.5	24	2.5
12	75	79	44	33	8	5	6.1	25	2.7
13	73	72	52	34	12	8	4.8	22	3.1
14	78	74	43	27	12	9	4.6	15	2.7
15	84	65	41	28	11	4	3.1	17	2.4
Median (maximum, minimum)	75 (109, 59)	74 (80, 63)	55 (75, 41)	37 (57, 27)	12 (14, 7)	5 (9, 2)	6.5 (14.4, 3.1)	26 (47, 15)	2.6 (3.3, 1.7)

*PG* pressure gradient, *LVEF* left ventricular ejection fraction, *PAP* pulmonary arterial pressure, *PAWP* pulmonary artery wedge pressure, *RAP* right atrial pressure, *PVR* pulmonary vascular resistance, *TPG* transpulmonary pressure gradient, *CI* cardiac index

### Clinical outcome after diagnosis of pre-capillary PH

All 15 patients were followed until December 31, 2015 in Toujinkai Hospital. Of 15 patients, 11 died of heart failure: their mean survival time was 26.4 ± 21.0 months. Closure of vascular access had been done in 6 of 15 patients immediately after the diagnosis; however, 5 of the patients died thereafter (Table 3). Differences in clinical features between patients with or without heart failure death are shown in Table 4. Closure of vascular access did not affect the incidence of heart failure death. Although mean systolic PGs in tricuspid valve at the onset of PH did not differ between the groups with or without heart failure death, those at 3 months after the onset were higher in patients with heart failure death than in those without. Administration rate of ETAs was 100 % in patients without heart failure death, and 64 % in those with heart failure death.

**Table 3:** Treatment and outcome after diagnosis of pulmonary hypertension

Patient number	Closure of vascular access (1 = yes, 0 = no)	Endothelin receptor antagonists	Heart failure death (1 = yes, 0 = no)	Survival after the onset (months)
1	1	0	1	6
2	0	Bosentan 250 mg/day	0	96
3	1	Bosentan 125 mg/day	1	18
4	1	Bosentan 187.5 mg/day	0	117
5	0	Bosentan 187.5 mg/day → Ambrisentan 5 mg/day	1	61
6	1	Bosentan 125 mg/day	1	18
7	0	0	1	13
8	0	Bosentan 125 mg/day → Ambrisentan 2.5 mg/day	1	58
9	0	Bosentan 250 mg/day → Ambrisentan 5 mg/day	1	44
10	1	0	1	8
11	0	Bosentan 125 mg/day → 0	1	49
12	0	Ambrisentan 2.5 mg/day	0	20
13	0	Bosentan 62.5 mg/day → Macitentan 5 mg/day	0	16
14	1	Ambrisentan 2.5 mg/day	1	12
15	0	0	1	3
	6/15	11/15	11/15	*18 (117, 3)

* Median (maximum, minimum)

**Table 4:** Differences in clinical features between patients with or without heart failure death

	Heart failure death (*n* = 11)	Survived (*n* = 4)	*P*
Age, years	71 (83, 53)	58 (76, 46)	0.280
Dialysis duration, months	159 (436, 0)	160 (519, 6)	0.979
Diabetes mellitus, *n* (%)	1/11(9)	2/4 (50)	0.255
Female gender, *n* (%)	9/11 (82)	3/4 (75)	0.817
Body mass index, kg/m^2^	18.9 (24.6, 17.7)	21.0 (26.3, 17.7)	0.661
Systolic blood pressure, mmHg	76 (88, 56)	80 (82, 72)	0.753
Duration after making vascular access, months	135 (400, 3)	4 (4, 6)	0.104
Closure of vascular access, *n* (%)	5/11 (45)	1/4 (25)	0.516
Systolic pressure gradient in tricuspid valve at the onset, mmHg	84 (109, 59)	72 (75, 68)	0.226
Systolic pressure gradient in tricuspid valve 3 months after the onset, mmHg	84 (120, 24)	18 (28, 8)	0.010
Left ventricular ejection fraction (%)	74 (78, 63)	76.5 (80, 72)	0.226
Systolic PAP, mmHg	55 (75, 41)	55 (75, 44)	0.949
Diastolic PAP, mmHg	23 (36, 15)	30 (40, 20)	0.280
Mean PAP, mmHg	37 (50, 27)	39 (57, 33)	0.571
RAP, mmHg	6 (9, 2)	4.5 (8, 4)	0.661
PVR, Woods units	6.5 (14.4, 3.1)	7.8 (9.5, 4.8)	0.851
PAWP, mmHg	12 (14, 7)	11 (12, 8)	0.226
TPG, mmHg	25 (36, 15)	29 (47, 22)	0.351
CI, l/min/m^2^	2.5 (3.0, 1.7)	2.9 (3.3, 2.3)	0.412
Blood hemoglobin, g/l	109 (133, 95)	121 (134, 107)	0.226
Serum calcium, mmol/l	2.3 (2.4, 2.1)	2.2 (2.3, 2.0)	0.571
Serum inorganic phosphate, mmol/l	1.5 (1.8, 0.7)	1.4 (1.7, 1.2)	0.753
Serum albumin, g/l	35 (39, 28)	34 (37, 31)	0.961
Serum C-reactive protein, mg/l	2.5 (6.3, 0.2)	2.1 (6.4, 0.7)	0.949
Endothelin receptor antagonists, *n* (%)	7 (64)	4 (100)	0.038

Continuous variables are expressed as median value (maximum, minimum)

*PAP* pulmonary arterial pressure, *PAWP* pulmonary artery wedge pressure, *RAP* right atrial pressure, *PVR* pulmonary vascular resistance, *TPG* transpulmonary pressure gradient, *CI* cardiac index

### ETAs and heart failure death

**Fig. 2:**
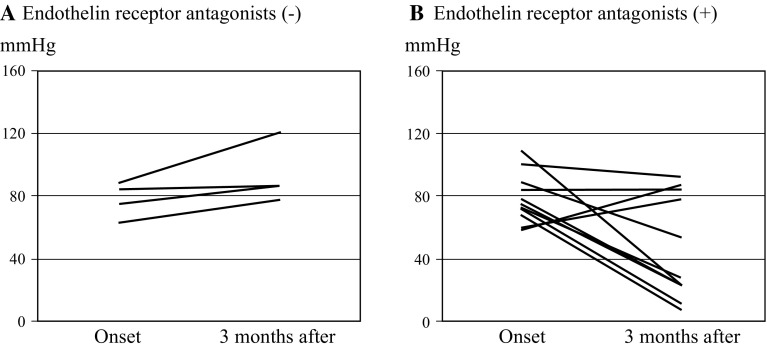
Changes in systolic pressure gradients in tricuspid valve at onset of pulmonary hypertension and at 3 months after the onset. Mean systolic pressure gradients in tricuspid valve tended to increase in 4 patients without endothelin receptor antagonists (**a**) (77.5 ± 11.1 versus 92.5 ± 18.7 mmHg, *P* = 0.097), whereas those decreased in patients administered with endothelin receptor antagonists (**b**) (78.8 ± 15.7 versus 46.8 ± 32.7 mmHg, *P* = 0.015)

**Fig. 3:**
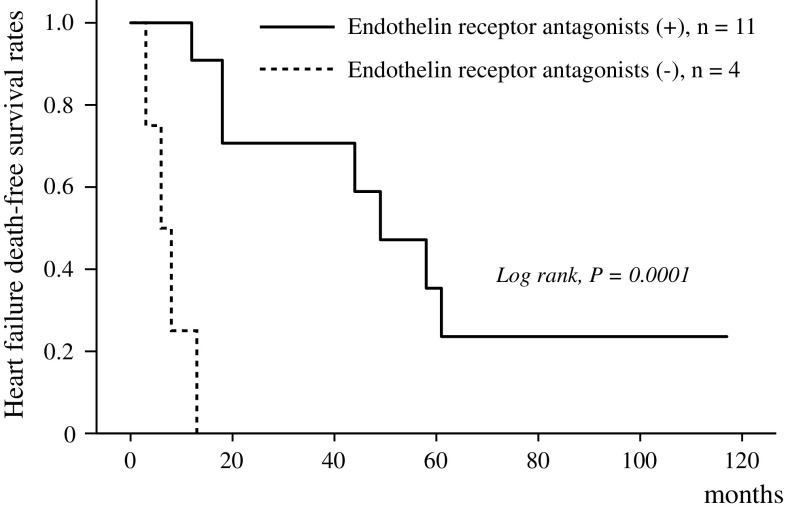
Heart failure death-free survival rates in patients with or without endothelin receptor antagonists. Mean survival time in patients without endothelin receptor antagonists was 7.5 ± 2.1 months. Heart failure death-free survival rates at 1 and 3 years were 100 and 71 % in patients with endothelin receptor antagonists, whereas they were 25 and 0 % in patients without

Of 15 patients with pre-capillary PH, 1 patient (patient number 1) died before the appearance of an ETA, and three patients (patient number 7, 10, 15) tried ETAs, but could not endure the administration of the ETAs because of severe hypotension during hemodialysis. In consequence, ETAs were used in 11 patients (Table 3). Baseline clinical features did not significantly differ between patients with or without ETAs (Table 5). Bosentan was used in 8 patients at the doses of 62.5–250 mg/day; however, bosentan was changed to ambrisentan in 3 patients and to macitentan in 1 patient, because the PH and right-sided heart failure worsened. One patient (patient number 11) stopped administration of bosentan 3 years after administration, because PH was improved; this patient died of heart failure 13 months after stopping bosentan. Ambrisentan was used from the start in 2 patients at the dose of 1.25–2.5 mg/day. Macitentan was changed from bosentan and used at the dose of 1.25–5 mg/day in 1 patient (patient number 13). These doses of ETAs were determined according to the blood pressure levels, including while undergoing hemodialysis. Mean systolic PGs in tricuspid valve at 3 months after the onset of PH decreased in patients administered with ETAs, whereas those tended to increase in 4 patients without an ETA (Fig. 2). In the Kaplan–Meier analysis, heart failure death-free survival rates were significantly higher in patients with ETAs than in those without (Fig. 3). Mean survival times at the point of December 31, 2015 were 57.3 ± 12.1 months in patients with ETAs and 7.5 ± 2.1 months in those without. In the Cox hazard analysis, heart failure death was associated positively with systolic PG in tricuspid valve at 3 months after the onset [1 mmHg: hazard ratio, 1.03; 95 % confidence interval (CI), 1.01–1.06; *P* = 0.013] and inversely with administration of an ETA (hazard ratio, 0.04; 95 % CI, 0.01–0.38; *P* = 0.005), and tended to be associated with cardiac index (1 l/min/m^2^: hazard ratio, 0.27; *P* = 0.055) or age (1 year: hazard ratio, 1.06; *P* = 0.098).

**Table 5:** Differences in baseline clinical features between patients with or without endothelin receptor antagonists (ETAs)

	ETAs (+) (*n* = 11)	ETAs (−) (*n* = 4)	*P*
Age, years	70 (76, 46)	71 (83, 62)	0.571
Dialysis duration, months	214 (519, 0)	147 (396, 4)	0.661
Diabetes mellitus, *n* (%)	3/11 (27)	0/4 (0)	0.082
Female gender, *n* (%)	9/11 (82)	3/4 (75)	0.817
Body mass index, kg/m^2^	19.5 (26.3, 17.7)	18.6 (23.3, 18.1)	0.489
Systolic blood pressure, mmHg	76 (88, 56)	80 (84, 68)	0.661
Duration after making vascular access, months	6 (250, 3)	151 (400, 3)	0.412
Closure of vascular access, *n* (%)	4/11 (36)	2/4 (50)	0.694
Systolic pressure gradient in tricuspid valve at the onset, mmHg	75 (109, 59)	80 (88, 63)	0.949
Left ventricular ejection fraction (%)	74 (80, 63)	73 (76, 65)	0.489
Systolic PAP, mmHg	54 (75, 43)	64 (75, 41)	0.571
Mean PAP, mmHg	36 (57, 27)	41 (50, 28)	0.489
RAP, mmHg	5 (9, 2)	6 (8, 4)	0.949
PVR, Woods units	6.2 (9.5, 4.6)	9.8 (14.4, 3.0)	0.343
PAWP, mmHg	12 (14, 7)	12 (14, 11)	0.489
TPG, mmHg	25 (47, 15)	28 (36, 17)	0.661
CI, l/min/m^2^	2.7 (3.3, 1.7)	2.1 (2.8, 1.7)	0.138
Blood hemoglobin, g/l	113 (134, 97)	107 (119, 95)	0.343
Serum calcium, mmol/l	2.2 (2.3, 2.0)	2.4 (2.4, 2.2)	0.056
Serum inorganic phosphate, mmol/l	1.4 (1.8, 0.9)	1.5 (1.8, 0.7)	0.661
Serum albumin, g/l	34 (39, 28)	36 (37, 31)	0.661
Serum C-reactive protein, mg/l	3.1 (6.4, 0.2)	2.4 (2.7, 0.4)	0.489

Continuous variables are expressed as median value (maximum, minimum)

*PAP* pulmonary arterial pressure, *PAWP* pulmonary artery wedge pressure, *RAP* right atrial pressure, *PVR* pulmonary vascular resistance, *TPG* transpulmonary pressure gradient, *CI* cardiac index

## Discussion

In the present investigation, idiopathic pre-capillary PH was recognized in 0.75 % of ESKD patients. Although the prevalence of PH seems to be low compared with the previous studies [4–10], it is extremely high compared with that of group 1 PAH without ESKD [2, 3]. Furthermore, the prognosis of idiopathic pre-capillary PH in ESKD patients would be worse than that in the previous reports; the survival at 1 year after diagnosis was over 70 % [7, 9]. Since echocardiography was used for diagnosis of PH in these studies, patients with conditions, such as volume overload or diseases, other than idiopathic pre-capillary PH would have been included, and this might have affected the results. It would be important to recognize that, without detection, diagnosis, and treatment, many ESKD patients with idiopathic pre-capillary PH are at risk of heart failure death.

Vascular access, such as AVF or arteriovenous graft, increases cardiac output and preload on the right heart chambers, and contributes to the increase in pulmonary arterial flow and pressure [16]. These hemodynamic changes in pulmonary artery stimulate the production of inflammatory cytokines and growth factors, such as fibroblast growth factor, with concomitant pulmonary angiotensin-converting enzyme activation [17, 18]. These local changes in pulmonary arterial walls cause abnormal proliferation of smooth muscle cells until fibrosis. On the other hand, lower activation of nitric oxide synthesis and increased synthesis of endothelin-1 cause endothelial dysfunction and proliferation, leading to obliteration of pulmonary vessels [19]. Since PH occurred within 6 months after making vascular access in 8 of 15 patients in this study, increased pulmonary blood flow by vascular access might have played a role in the pathogenesis of PH in this population. Some studies showed that PAP and cardiac output reduced after ligation or compression of the AVF [4, 20, 21]. However, closure of vascular access was not associated with inhibition of heart failure death in our study. Further investigation is needed to clarify whether closure of vascular access is beneficial for the improvement of pre-capillary PH in ESKD patients.

Patients without ETAs died of heart failure from 3 to 13 months after the diagnosis, whereas those with ETAs survived longer. Although the administration of an ETA reportedly improved exercise capacity, dyspnea, and hemodynamic parameters, such as PAP, PVR, or cardiac index in a meta-analysis of 12 randomized trials on group 1 PAH [22], no study has been reported on the effect of ETAs on PH in ESKD patients. Plasma concentration of endothelin-1 reportedly increased in hemodialysis patients compared with normal persons [16]. Endothelin-1 may play a role in the pathogenesis of idiopathic pre-capillary PH in ESKD as well as in group 1 PAH. Endothelin-1 has two types of receptors: type A and B. Among the ESAs used in our study, bosentan and macitentan are non-selective dual action receptor antagonists, and ambrisentan is a selective antagonist of type A. We could not determine which types of ETAs would be effective for idiopathic pre-capillary PH in ESKD, because of the small sample size in our study. In a previous study [23], bosentan needed no adjustment of the dosing regimen in administration to ESKD patients. However, it would be necessary to administer the ETA beginning with a small amount when using ETAs for PH in ESKD, particularly in hemodialysis patients, as not to further decrease blood pressure. It is also important to increase the dose of the ETA slowly, according to the blood pressure levels. Furthermore, we may have to pay attention to the occurrence of congestive heart failure when we administer ETAs to ESKD patients with preserved renal function, because blockade of endothelin receptor reportedly causes water and salt retention, especially in patients with renal insufficiency [24, 25].

This study had several significant limitations. This was a retrospective, observational study performed in a single center. Use or non-use of an ETA and types of ETAs were not randomized among the patients. Because the sample size was very small, statistical results might not be reliable. A multi-center, randomized, and prospective trial would be needed to confirm the effects of ETAs on pre-capillary PH of ESKD patients. RHC was performed to diagnose pre-capillary PH, but was not undergone repeatedly to ascertain the clinical course or the effect of ETAs in all patients. We had confirmed the heart failure death, but did not evaluate the changes in exercise capacity or symptoms, such as dyspnea. We used the value of systolic PGs in tricuspid valve ≥35 mmHg for screening of pre-capillary PH. We might have missed some patients with PH in this study, since the screening value of systolic PGs in tricuspid valve is ≥33.64 mmHg (≥2.9 m/s) in the 2015 ESC/ERS guidelines [1].

We believe that the results of this study showed four important findings: first, the prevalence of idiopathic pre-capillary PH in ESKD is likely to be high compared with that of group 1 PAH; second, making of a vascular access might be partly involved in the pathogenesis of pre-capillary PH in ESKD. However, it is uncertain whether closure of vascular access after the onset of PH is effective for the improvement of PH or survival; third, the prognosis of idiopathic pre-capillary PH in ESKD seems to be poor compared with that of group 1 PAH; and fourth, administration of ETAs has a potential to improve the survival by inhibiting heart failure death. It is important for clinical nephrologists to recognize that pre-capillary PH is one of the causes of right-sided heart failure, and that the early detection and treatment of pre-capillary PH could contribute to further improving the prognosis of ESKD patients.
